# Feeding of a Scleractinian Coral, *Goniopora columna*, on Microalgae, Yeast, and Artificial Feed in Captivity

**DOI:** 10.3390/ani11113009

**Published:** 2021-10-20

**Authors:** De-Sing Ding, Wei-Ting Sun, Chih-Hung Pan

**Affiliations:** 1 Ph.D. Program of Aquatic Science and Technology in Industry, College of Hydrosphere Science, National Kaohsiung University of Science and Technology, No. 142, Haijhuan Rd., Nanzih District, Kaohsiung 811213, Taiwan; 2 Department and Graduate Institute of Aquaculture, National Kaohsiung University of Science and Technology, No. 142, Haijhuan Rd., Nanzih District, Kaohsiung 811213, Taiwan; 1051537103@nkust.edu.tw (W.-T.S.); chpan@nkust.edu.tw (C.-H.P.)

**Keywords:** feeding, growth, digestive enzymes, *Goniopora columna*, scleractinian coral

## Abstract

**Simple Summary:**

Coral aquaculture is an innovative and sustainable aquaculture industry. Coral husbandry can address ecological environment conservation needs and industrial demand for corals. Many previous studies have confirmed that corals also belong to heterotrophic organisms. Heterotrophic feeding is essential for overcoming nutrient deficiency. The preliminary results of this study indicate that *Goniopora columna* have high levels of proteases, and artificial feeds containing high protein can be used for feeding during aquaculture, which can increase the growth rate. In conclusion, we have initially explored that *Goniopora columna* will have better growth by feeding artificial PUFA rich in animal protein. In addition, the best feeding time is 6:00–12:00 in the morning, when there is better digestion and absorption. It is hoped that this research will be helpful to the development of coral aquaculture in the future.

**Abstract:**

Nutritional requirements are critical in the process of coral aquaculture. In addition to energy from symbiotic algae, corals obtain sufficient nutrition through heterotrophic feeding. Microalgae and yeast are commonly used as nutritional supplements for many aquaculture organisms. In addition, if artificial feed can match or improve upon the nutritional supplementation provided by microalgae and yeast in the case of *G. columna*, then feeding this coral would be markedly easier. Hence, this article preliminarily discusses feeds suitable for *G. columna*. In this study, artificial PUFA rich in animal protein (R), *Saccharomyces cerevisiae*, *Isochrysis galbana* tml, and *Nannochloropsis oculate* were fed to *G. columna* at quantities of 5% and 10% of body weight. Growth, survival, body composition, and digestive enzymes were assessed. Regarding body composition, the coral’s protein content is higher than that of carbohydrate or fat; thus, evaluating the heterotrophic nutrition of *G. columna* by using protein absorption is appropriate. The protease content is also high in digestive enzymes. Protein content, protease activity, and specific growth rate were significantly higher in the R group than in other groups. The number of polyps in the groups fed R at 5% and 10% of body weight increased by 40.00 ± 2.43 and 47.33 ± 0.89 number, respectively, significantly greater increases than those achieved in the other groups (*p* < 0.05). Changes in body composition and digestive enzymes over a 24-h period were compared to determine the optimal feeding time. Protein content and protease activity increased markedly between 6:00 and 12:00. The experimental results suggest that R can improve the activity of *G. columna* digestive enzymes and their protein and lipid content in body tissue, shorten the cultivation time, and enhance the profitability of coral aquaculture.

## 1. Introduction

Coral reefs are considered the tropical rainforests of the sea because of their rich species diversity [1,2]. However, coral reefs are deteriorating at an alarming rate due to climate change, environmental pollution, and destructive fishing practices. Coral bleaching, which can lead to species extinction, is increasingly occurring. Coral husbandry can simultaneously address environmental conservation needs and the industrial demand for corals. According to the Convention on International Trade in Endangered Species of Wild Fauna and Flora (CITES), the total trade volume of *Goniopora* sp. from 2010 to 2020 accounted for 18% of the trade volume of all corals, amounting to US $7.2 million (CITES, 2021). Although the species has noteworthy ornamental value, research has indicated that the mortality of *Goniopora* sp. in aquariums is as high as 95% [3]. Aquaculture of coral offers an alternative to wild harvesting for the ornamental trade and shows considerable promise for restoring reefs and preserving biodiversity [4]. *Goniopora columna* is a scleractinian coral native to the western Pacific Ocean and the eastern and western Indian Ocean [2]. This reef-associated species, commonly known as a flowerpot coral, is primarily found in intertidal coral reefs and often forms large groups in muddy waters. Its corallites are >5 mm in diameter, and its polyps are large, fleshy, and long. Tubular creatures with conical projections in the center of their tentacles, they live in seas rich with nutrients and can supplement heterotrophic nutrition with polyp hunting. *G. columna* are considered a heterotrophic coral. The autotrophic and heterotrophic nutrition of corals and the relative importance of these sources in their energy balance have long been recognized as key biological and ecological concerns. *G. columna* has both ecological and ornamental roles. Therefore, their reefs must be efficiently restored and protected; this can be achieved by determining the optimal culture conditions for *G. columna*. In addition to light and water flow, the organic matter or feed in the environment plays a crucial role in coral aquaculture [5].

Corals are heterotrophic organisms; heterotrophic feeding is essential for them if they are to avoid nutrient deficiency. Corals exhibit several modes of feeding and acquire energy from multiple sources [6]. In addition to obtaining nutrients from symbiotic algae, corals can feed on marine plankton, which provide essential amino acids and other nutrients not obtainable from symbiotic algae [6,7,8,9]. Numerous studies have reported that many species of corals are also active heterotrophs, ingesting bacteria, particulate organic matter, and even dissolved nutrients from water and mesozooplankton. Heterotrophy accounts for up to 66% of the fixed carbon incorporated into the skeleton of a coral and meets 15–35% of the daily metabolic requirements, contributing to the metabolic activity associated with zooxanthellae [10,11,12,13,14,15,16,17,18,19,20] Regarding the feeding habits of the coral *Montastraea cavernosa*, the larvae of copepods, amphipods, nemerteans, turbellarians, polychaetes, nematodes, appendicularians, salps, decapods, and cirripedes have been discovered in the coral body and planktonic algae [21] Studies have suggested that corals are polytrophic in nature [22].

To date, only a few studies have explored the nutrition and digestion of corals as well as whether feeding is required to induce a growth rate conducive to large-scale artificial cultivation. In coral aquaculture, asexual fragmentation is often used for propagation. The partitioned coral colony tends to be small, and the nutrients provided by coral symbiotic algae may be insufficient. Therefore, hunting heterotrophic nutrients to promote growth and accelerate tissue repair is necessary. The growth and survival of artificially cultured corals are affected by the quality of available nutrient sources [23]. Heterotrophic feeding enhances the growth, calcification rate, and photosynthesis rate of coral and even affects the symbiotic zooxanthellae [6,24,25,26,27,28,29]. Obtaining plankton from the ocean to feed coral in an aquarium is unsustainable and economically inefficient. A common initial diet in aquaculture comprises microalgae and yeast. Microalgae contain oil and unsaturated fatty acids, which can improve the nutrition, growth, and survival rate of fish fry. Yeast can provide nutritional supplement to promote the growth of some marine life and has also been proven to strengthen nonspecific immune responses [30,31]. Until now, no studies have evaluated the effects of microalgae, yeast, and artificial coral feed on *G. columna* nutrition. This study involved experiments to determine such effects of microalgae, yeast commonly used in aquaculture, and artificial coral feed. By analyzing the resultant body composition, changes in digestive enzymes, and growth of *G. columna*, we evaluated the suitability of these feeds and the optimal feeding time to indicate how the growth rate of *G. columna* during cultivation can be improved.

## 2. Materials and Methods

### 2.1. Biological Materials

*G. columna* (total of 100 colonies) was obtained from Coral King Coral Farm (Kaohsiung, Taiwan), a CITES-legal coral farm (no. FTS507W0153796), and were cultured in an aquarium tank (60 × 35 × 30 cm) by using a recirculating filtered seawater system. An HME Block 2 Series frame with purple light-emitting diode lights (400–430 nm; photosynthetically active radiation [PAR] 71.03 ± 0.21 μmol m^−2^s^−1^) was set 30 cm above the glass aquarium’s water surface. The PAR was detected using an Apogee Instruments MQ-510 underwater quantum meter (USA). The water quality was monitored daily during the experiment and maintained at a safe level. When corals were moved to a tank, they immediately produced abundant mucus to protect themselves from bacterial infection; therefore, pump-generated water flow was used to remove mucus from the corals’ surface and prevent hypoxia, which would affect the corals’ physiological metabolism, and tissue necrosis caused by the slimy covering [9]. Therefore, a water pump should be installed in aquariums to remove the mucus on the surface of corals by using water flow. After the corals had adapted to their environment, they were artificially propagated through fragmentation. In this study, after 2 months of acclimation and self-repair, healthy corals were segmented into groups, with each colony containing five polyps; then, they were fixed onto porous foundation stones by using coral glue. After approximately 72 h of tissue repair, the polyps were fully extended (to assess the tissue repair) and the experiment was started. All experiments were performed triplicate, and thus each group involved a total of 30 colonies.

### 2.2. Experiment One: Feed Composition and Feeding

#### 2.2.1. Feed Source

The R diet contained a mixture of intact and hydrolyzed marine and terrestrial ingredients (commercial-in-confidence formulation, details not provided). The S, I, and N feeds were employed as plant feeds in this study. Microalgae in stock cultures were grown in a 2000 mL glass conical flask containing liquid Walne medium. High-temperature, high-pressure sterilization was applied (121 °C for 30 min). The culture was then cooled to 26 °C, and a fluorescence lamp with 12L/12D-h light–dark photoperiod was used for irradiation. Subcultures were then formed by transferring 400 mL of solution with viable microalgae cells to new medium every 1–2 months. The nutritional composition of all feeds is shown in Table 1.

#### 2.2.2. Coral Feeding

In the coral feeding assessment, microscopic examination of the corals 1 h after feeding revealed the presence of microalgae and yeast in the corals’ body cavities, but the R formulation could not be observed under a microscope because this commercial feed was a liquid. This study evaluated the coral feed’s effects on nutrient uptake with reference to changes in the protein content of *Psetta maxima* L. levels by feeding plant proteins in accordance with the experimental method of [32]. The feeding density of microalgae and yeast was 5 − 6 × 10^5^ cells/mL. When the density was too high, sterilized seawater was used for dilution. Microalgae, yeast, and R are all liquid, so 10% (*w/v*) of coral tissue and skeletal dry weight are used for feeding. Therefore, the protein, lipid, and carbohydrate content of the experimental corals (in their body composition) were compared with those of the unfed control group to determine whether food had been ingested. Protein, lipid, and glucose analysis was performed 1 h after feeding.

#### 2.2.3. Analysis of Coral Body Composition and Feed

In this study, in addition to analyzing the body composition analysis of the corals, feed analysis was conducted for data comparison. Each group of feeds was centrifuged to obtain 1 mg as a sample for analysis. G. columna were sonicated and protein concentrations were measured using a Bradford protein assay kit (Ameresco, Solon, OH, USA) with bovine serum albumin as a protein standard. Lipids content analysis was performed using the soxhlet extractor methods [33]. The total lipid weight was determined (±0.0001 g), and the derived weight values were converted into micrograms (1 g = 1 × 10^6^ µg). Glucose detection is based on the Enzymatic, Colorimetric method (GOD/PAP) with glucose oxidase, and 4-aminoantipyrine [34,35].

### 2.3. Experiment Two: Effects of Different Diets on Body Composition, Digestion Enzyme, Growth and Survival of Coral

#### 2.3.1. Experimental Conditions

In this study, artificial polyunsaturated fatty acid (PUFA) rich in animal protein (R; i.e., a formulated diet combining animal protein and sodium alginate with probiotics), *Saccharomyces cerevisiae* (S), *Isochrysis galbana* tml (I), and *Nannochloropsis oculata* (N) were used as the feeds for the experimental groups; an unfed control group (C) was also included in the study. The feeding density of microalgae and yeast was 5 – 6 × 10^5^ cells/mL, and the feeding amount was either 5% (denoted 5) or 10% (10) of the coral’s body weight (*w/v*), such that the experimental groups are represented as R(5), S(5), I(5), N(5), R(10), S(10), I(10), and N(10) and the control group as C, each with three respective replicates. The diet formula was improved after reference to the work of [36,37,38]. We have studied this feed and hope to successfully conduct large-scale *G. columna* culture. Each treatment group had triplicate (with 10 colonies each) and a total of 30 colonies. The water quality conditions were summarized in Table 2. The experiment lasted for eight weeks. After the experiment, the initial and final weights were measured to calculate the specific growth rate (SGR), body composition, digestion enzyme, zooxanthellae, and chlorophylla.

#### 2.3.2. Coral Feeding

After shaking the microalgae and yeast evenly, 20 μL was absorbed by a micropipette (adjustable air-displacement pipette M25) and then used to calculate the number of microalgae and yeast cells with a hemocytometer [26]. The cultured microalgae and yeast were first counted by hemocytometer, and then diluted with sterilized seawater to the desired feeding density. The feeding density of microalgae and yeast was 5 – 6 × 10^5^ cells/mL. Microalgae, yeast, and R were liquid and fed 5% and 10% (*w/v*) of coral tissue and skeletal dry weight per day. Feeding occurred daily at 8:00, except in the case of the unfed control group.

#### 2.3.3. Determination of Coral Growth and Polyp Count

Coral growth was determined on the basis of total weight and polyp count as described by [39,40]. We followed Hii et al. [6] by sampling and analyzing the different feed groups 1 h after feeding. We measured the tissue and skeletal dry weight. Algae were brushed and the coral surfaces were dusted off and then placed on a plastic petri dish. Subsequently, a coral’s weight was measured using an electronic balance. *G. columna* has a large polyp that can be observed directly with the naked eye. Calculations were made once a week, and photographs to record the number of new polyps were taken using a Canon EOS 750D camera. The SGR of the coral was measured using the following formula:

(1) $$\mathrm{SGR}\;(\%\;\mathrm{day}{-}^{1})=\left(\frac{In\left(wf\right)-In\left(wi\right)}{\Delta t}\right)\times 100$$

where *wi* is the initial weight of the coral (g), *wf* is the final weight of the coral (g) and Δt is the number of experimental days.

The tissue and skeletal dry weight and number of polyps were measured every 7 days. SGRs as mean values with standard deviations (SDs) were calculated after the experiment. After the experiment, to calculate the survival of the corals, the disappearance of polyps was considered to indicate their death. The survival rates, expressed as means (%) with SDs, were calculated using the following formula:

(2) $$\mathrm{survival}\;\mathrm{rate}\left(\%\right)=\frac{\left(\mathrm{number}\;\mathrm{of}\;\mathrm{final}\;\mathrm{living}\;\mathrm{specimens}\right)}{\;\left(\mathrm{number}\;\mathrm{of}\;\mathrm{initial}\;\mathrm{specimens}\right)}\times 100$$

#### 2.3.4. Analysis of Zooxanthellae Density and Chlorophyll a

After the eight-week experiment, coral tissues were homogenized and zooxanthellae density was calculated according to Titlyanov et al. [26] method. Chlorophyll a concentration was determined by Hitachi U-2000 spectrophotometer at 630 nm and 664 nm in reference to Levy et al. [41] and Titlyanov et al. [26] method. Calculated using an equation developed by Jeffrey & Humphrey [42], the value represents how many micrograms of chlorophyll a there are per gram of coral tissue.

#### 2.3.5. Analysis of Coral Body Composition and Digestive Enzymes

For coral body composition test, please refer to materials and methods 2.2.3. Enzyme extraction was performed using the method of Sun et al. [43]. Protease and lipase extractions were performed using 10 mM sodium citrate buffer (pH 7.0) in a cold environment. Each coral was first rinsed in buffer solution and then added to 10 times the volume of buffer solution, placed on ice for grinding, and subsequently centrifuged (10,000× *g*) at 4 °C for 10 min. Thereafter, the supernatant was collected and stored at −20 °C. Protease content was analyzed using the method of Sun et al. [43], which involved adding 1 mL of casein to 0.5 mL of enzyme extract, incubating the mixture for 15 min, and then adding 1.5 mL of 10% trichloroacetic acid. After centrifuging (6000× *g*) at 4 °C for 10 min, the supernatant was collected and 5 mL of 0.55 M Na_2_CO_3_ and 1 mL of Folin phenol-staining reagent were added. The absorbance value at 680 nm was observed. Lipase content was analyzed using the method of Borlongan [44]. To 1.5 mL of olive oil, 1.5 mL of Tris–HCl (0.1 M buffer, pH 8.0) and 1 mL of enzyme extract were added, and the solution was then shaken at 37 °C for 6 h. Thereafter, 95% alcohol was added to terminate the reaction, and thymolphthalein containing 0.9% alcohol was used as the indicator. The mixture was then titrated with 0.01 N NaOH until the solution turned brown. Amylase content was analyzed using the method of Bernfeld [45]. To 0.05 M phosphate buffer solution (pH 7.0), 1 mL of 2% (*w/v*) starch solution was added, and the mixture was maintained at 25 °C for 5 min. Then, the enzyme extraction was added and the mixture was left to react at 20–60 °C. Subsequently, 2 mL of dinitrosalicylic acid reagent was added before the reaction was stopped in a boiling water bath for 5 min and the mixture then cooled. The absorbance at 520 nm was measured, as per the maltose standard. Amylase activity was determined as maltose content per milligram of protein per minute. At the end of the experiment, means and SDs were calculated.

### 2.4. Experiment Three: Diurnal Change Analysis of Coral Body Composition and Digestive Enzymes

*G. columna* were fed in an aquarium (60 × 35 × 30 cm) containing a recirculating filtered seawater system. The photoperiod was set as 6/18-h light–dark. In this experiment, R was used for feeding, feeding at the same time at the beginning of the experiment, feeding 10% (*w/v*) of the tissue and skeletal dry weight of coral. Samples were obtained every 6 h, at 6:00, 12:00, 18:00, and 24:00. The corals’ body composition and digestive enzymes were analyzed after sampling (*n* = 30 colonies). The purpose of this study was to determine at which time of day corals should be fed to maximize digestion and absorption.

### 2.5. Statistical Analysis

Data were obtained from three independent experiments, and the final results are presented as means ± SDs. One-way analysis of variance and Duncan’s multiple range test were used to determine the statistical significance of *G. columna* nutrient composition, growth, survival, digestive enzyme activity, and body composition. A *p* value <0.05 was considered significant. All statistical analyses were performed using IBM SPSS version 20. These analyses were used evaluate the feeding efficiency and feed time of corals on different foods.

## 3. Results

### 3.1. Experiment One: Feed Composition and Ingestion

#### Effects of Feed on Nutrient Intake

Protein, lipid, and glucose analyses were performed 1 h after feeding (Table 3) and used to evaluate the changes in body composition of the corals after ingestion of feed compared with the unfed control group. After the *G. columna* were fed R, S, I, and N for 1 h, various changes in their composition were immediately observed; their protein content was 430.45 ± 12.30, 322.05 ± 11.22, 332.35 ± 13.12, and 325.43 ± 13.20 µg/mg, respectively. The R-fed groups contained significantly more protein (*p* < 0.05) than the other groups. The lipid content was higher in the I and N groups, but the differences compared with the other groups were significant. The S groups contained more glucose.

The R feed contained the most protein (76.67 ± 1.56 µg/mg), the I and N feeds contained the most lipids (32.00 ± 2.00 and 20.33 ± 1.56 µg/mg, respectively), and the S feed was the highest in glucose (29.00 ± 0.67µg/mg) (Table 1). Therefore, the results indicated that *G. columna* could increase some bodily nutrients through the intake of different feeds.

### 3.2. Experiment Two: Effects of Different Diets on Body Composition, Digestion Enzyme, Growth and Survival of Coral

#### 3.2.1. Effects of Feed on Growth and Survival of *G. Columna*

**Figure 1:**
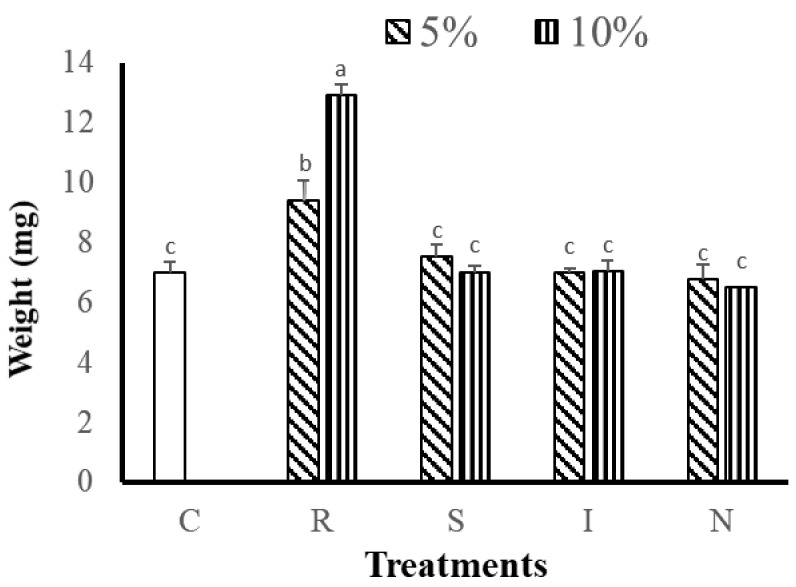
Tissue and skeletal dry weight of *G. columna* after 8 weeks of daily feeding with various aquaculture feeds. C: no feeding; R: artificial polyunsaturated fatty acid (PUFA) rich in animal protein; S: *Saccharomyces cerevisiae*; I: *Isochrysis galbana* tml; N: *Nannochloropsis oculata*. Different letters indicate significant differences among groups (*p* < 0.05). Values are expressed as means ± SDs (*n* = 30 colonies).

**Figure 2:**
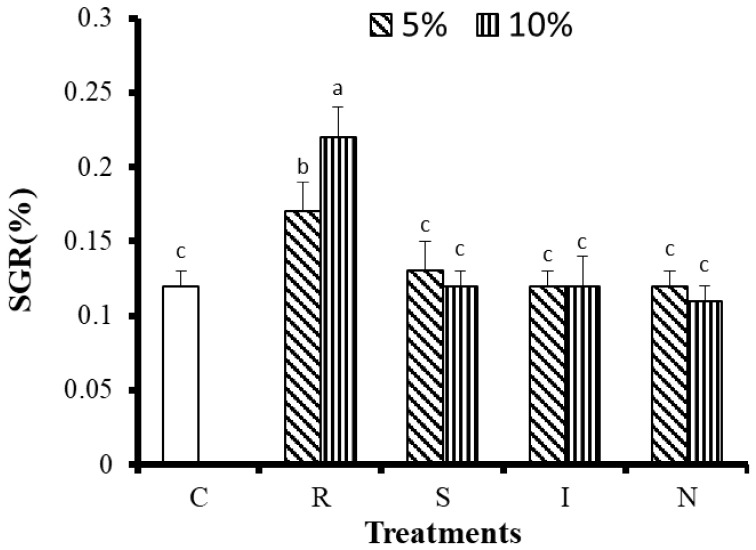
Growth of *G. columna* after 8 weeks of daily feeding. C: no feeding; R: artificial polyunsaturated fatty acid (PUFA) rich in animal protein; S: *Saccharomyces cerevisiae*; I: *Isochrysis galbana* tml; N: *Nannochloropsis oculata*. Different letters indicate significant differences among groups (*p* < 0.05). Values are expressed as means ± SDs (*n* = 30 colonies).

After 8 weeks of cultivation, the polyp numbers, tissue and skeletal dry weight measurements, and the SGRs of the corals were used to evaluate the growth of *G. columna*. As shown in Table 4, the numbers of polyps in the R(5) and R(10) groups were 40.00 ± 2.43 number and 47.33 ± 0.89 number, respectively, significantly higher than those in other groups fed 5% or 10% of their body weight (*p* < 0.05). The S(5) and N(5, 10) groups had the fewest polyps, with only 32 remaining after 8 weeks of culture. The R groups significantly outgrew all the other groups (*p* < 0.05). The *G. columna* exhibited stunted growth when fed the S, I, and N feeds. The changes in coral tissue and skeleton dry weights indicated that the R(10) group demonstrated the greatest tissue and skeletal dry weight gain (12.93 ± 0.4 mg; Figure 1). Figure 2 illustrates the SGR of the *G. columna* specimens. The SGRs of the R(5) and R(10) groups were 1.3 times those of the S, I, and N groups (for both 5% and 10% of body weight feeding). These results reveal that the selection of feed is crucial to *G. columna* growth. The R feed enhanced the growth of *G. columna*. In each group, the survival rate was 100%; no deaths were recorded. Thus, feeding with the R formulation effectively promotes *G. columna* growth.

#### 3.2.2. Influence of Different Feeds on Body Composition

To determine whether a selected feed affected the body composition of *G. columna*, the various feeds (R, S, I, and N) were given to the corals at quantities of either 5% or 10% of their body weight per day. After 8 weeks of cultivation, the body composition of the *G. columna* specimens was measured. Table 5 indicates that the protein content of the corals sustained with R feed was 474.01 ± 23.00 and 486.78 ± 36.41 µg/mg for those fed 5% and 10% of their body weight, respectively; this content was significantly higher than that of the other groups (*p* < 0.05). The lipid content of the R(5) and R(10) groups was 2.85 ± 0.13 and 3.02 ± 0.29 µg/mg, respectively, significantly higher than the values in the other groups (*p* < 0.05). There was no significant difference in glucose content among all treatments. According to body composition analysis, the protein content of *G. columna* is 166 and 370 times higher than that of lipid and glucose. Feeding high protein content of R helps *G. columna* to growth. According to the preliminary results, *G. columna* exhibits notable potential to absorb protein nutrients, which could boost their growth.

#### 3.2.3. Influence of Different Feeds on Digestive Enzymes

After the corals had been given different feeds (R, S, I, and N) for 8 weeks, their digestive enzyme activity was measured. As detailed in Table 6, the protease activity of the R(5) and R(10) groups was 1.57 and 1.67, 1.78 and 1.93, 1.69 and 1.71, and 1.51 and 1.52 times higher than that of S, I, N, and unfed control (C) groups, respectively. Similarly, the lipase activity of the R(5) and R(10) groups was 1.21 and 1.27, 1.29 and 1.27, 1.34 and 1.36, and 1.21 and 1.28 times higher than that of their counterpart S, I, N, and C groups. No significant differences in amylase activity were discovered among any of the groups. These results indicate that R feeding can enhance protease and lipase activities (but not amylase activity) in *G. columna*. Therefore, the intake of high-protein food is more conducive to nutrient absorption in *G. columna*.

#### 3.2.4. Influence of Different Feeds on Zooxanthellae and Chlorophyll a

By observing the changes of zooxanthellae and chlorophyll A, it can be judged that light energy or feed can provide nutrients to promote coral growth. As detailed in Table 4, no significant differences in zooxanthellae density (approximately 4.0 × 10^7^ cells m^−2^) or chlorophyll a concentration (approximately 54 µg cm^−2^) were noted among the groups fed different substances. Therefore, the experimental results showed that there was no significant increase in the number of zooxanthellae, but feeding R could promote the growth of coral.

### 3.3. Experiment Three: Coral Feeding Time Assessment

**Figure 3:**
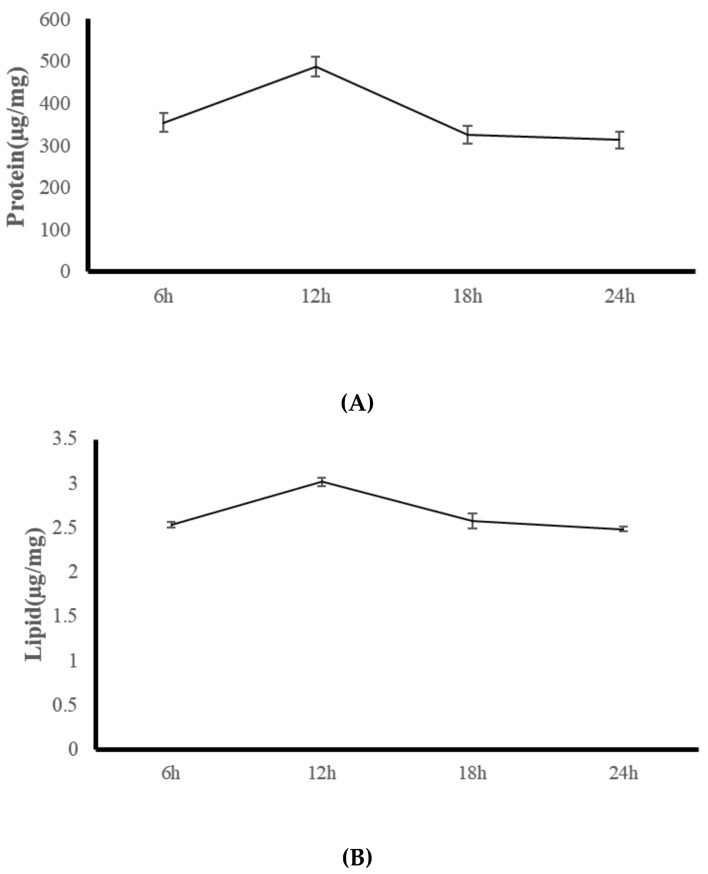

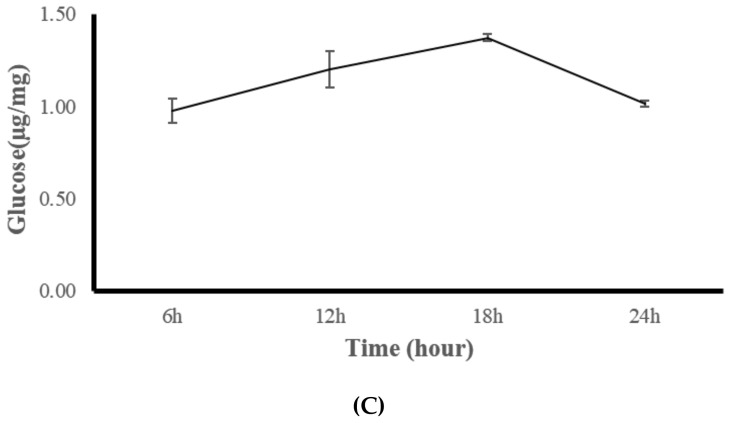
Changes in body composition of rich-animal-protein-fed (R-fed) *G. columna* over a 24-h period: (**A**) protein, (**B**) lipid, and (**C**) glucose content. Values are expressed as means ± SDs (*n* = 30 colonies).

The present study suggested that the artificial feed R enhances the growth, body composition, and digestive enzyme activity of *G. columna*. Changes in the body composition and digestive enzymes of *G. columna* were observed after feeding with R over a 24-h period. By measuring the changes in digestive enzymes and body composition, the feeding time most conducive to growth within coral aquaculture was evaluated. Figure 3 illustrates that the protein content of *G. columna* at 6:00, 12:00, 18:00, and 00:00 was 354.21 ± 21.64, 486.78 ± 23.12, 324.13 ± 21.03, and 321.49 ± 19.46 μg/mg, respectively. The protease activity at 12:00 was 1.37, 1.50, and 1.51 times higher than that at 6:00, 18:00, and 00:00, respectively. Lipid content was continually low (2.49–3.02 µg/mg). No significant differences in glucose content were observed among the four feeding times (1.02–1.38 µg/mg). As shown in Table 7, the protease activity at 6:00, 12:00, 18:00, and 00:00 was 153.25 ± 20.32, 385.67 ± 16.48, 285.15 ± 17.12, and 167.85 ± 19.35 U/mg protein, respectively. The protease activity at 12:00 was 2.52, 1.35, and 2.30 times higher than that at 6:00, 18:00, and 00:00, respectively. No significant differences were observed in the lipase or amylase activity among different feeding times. An in vivo comparison of the protease activity with protein content suggested that the protein content was lower when the protease activity was lower. The protein content and protease activity were both at their highest at 12:00 and both decreased in the evening to their lowest values at 00:00. Therefore, the experimental results suggest that the digestion and absorption of *G. columna* exhibit hour-to-hour changes across the daily cycle. The most suitable feeding time for *G. columna* culture is between 6:00 and 12:00, during which the digestive enzyme activity gradually increases and the absorption of nutrients from the feed is optimal.

## 4. Discussion

Among ornamental corals, *G. columna* is an industry favorite. The scarcity of data on the effects of various coral feeds and corals’ nutritional requirements renders feeding within coral aquaculture difficult [6]. *G. columna* cultures can be influenced by environmental factors, and quantitative feeding can enhance the growth of polyps. Our results revealed that *G. columna* gained effective sustenance from the R formulation. The R(10) group exhibited an increase in polyp numbers that was significantly greater than those of the other groups. No significant difference in daily growth rate was observed among the control group and S, I, and N experimental groups. At present, limited studies have investigated the nutrition of corals, and corals are usually cultured as small polyps. Because of the small size of these segmented corals, the coral symbiotic algae cannot provide their own nutrition, and nutritional sources are often lacking under the conditions of artificial cultivation, affecting the growth and survival rates of the cultivated corals [23]. In one study, feeding *Artemia salina* nauplii to *Galaxea fascicularis* resulted in a high feeding rate but did not improve coral growth, which may have been related to the body composition of the A. salina [6]. In addition, the caliber of *G. columna* is only 50–100 μm; hence, absorbing large feed organisms is impossible. Therefore, the size of a coral’s caliber should be considered during selection of feed. Among the feeds used in the present study, *S. cerevisiae*, *I. galbana* tml, and *N. oculata* could all be microscopically observed in the corals’ body cavities, and changes in body composition after feeding were also noted. We evaluated the nutrient absorption efficiency of corals on the basis of changes in body composition after providing *G. columna* with different feeds. The results suggested that the body composition of *G. columna* was affected by the feed given, with the experimental groups differing from the unfed control group. This may have been because different feeds contain different nutrients. Therefore, the results indicated that *G. columna* could increase some bodily nutrients through the intake of different feeds.

In addition to the feed quantity, the attractiveness of different feeds to *G. columna* may also affect the coral’s feeding habits. R is a liquid feed with particles approximately 1 μm in size. Thus, in addition to the composition of R, which was more suitable for *G. columna* absorption, the size of the feed particles may have been relevant. *G. columna* fed daily with R at 5% or 10% of its body weight exhibited enhanced protease activity and protein content, but the zooxanthellae density and chlorophyll a concentration remained unaffected. Overall, the results indicate that R feeding at a daily quantity of 5% or 10% of the coral’s body weight provides the optimal culture conditions for *G. columna*.

We analyzed the protein, lipid, and glucose content of the feed, as shown in Table 6. The protein content was highest in the R feed (76.67 ± 1.56 µg), I had the highest fat content, and S had the highest glucose content. *G. columna* body composition analysis indicated that R contained the most protein and the least carbohydrates. The protein content and growth efficiency were higher in the R-fed groups than in the other groups. Heterotrophic feeding is essential for corals to overcome any nutrient deficiency because nitrogen, phosphate, vitamins, and other trace elements are not synthesized through autotrophic feeding [6]. Raz-Bahat et al. (2017) [46] reported that the actinopharynx and mesenterial filaments are pivotal digestive systems in stony corals, and consideration of the digestive enzymes and chymotrypsinogen is crucial to elucidating the digestive system functionality of Stylophora pistillata. Therefore, corals can obtain nutrition through ingestion of food. Most stony corals can obtain 40–95% of base energy through the host [47,48]. Corals are also opportunistic heterotrophic feeders, exploiting several trophic pathways simultaneously [49]. Corals are known to range between the activities of a primary herbivore, producer, carnivore, detritivore, and consumer of dissolved nutrients [20,49]. These heterogeneous roles provide corals with myriad nutrients, such as phosphorus and alternative forms of lipids not provided by photosynthesis [20]. Our results suggest that feeding *G. columna* with R leads to considerable growth; by contrast, feeding it microalgae and yeast does not assist the growth of *G. columna*.

Yeast is a common live feed in aquaculture [50,51]. Different yeast cell walls have different components (e.g., β-glucan and oligosaccharide), and are commonly used as in fish diets or to replace fish meal [31,52,53,54,55]. Brewer’s yeast can replace 50% of the fish meal content in *Dicentrarchus labrax* feed [31]. Feeding 25% brewer’s yeast can replace casein and improve the growth and feed conversion rate of rainbow trout (*Oncorhynchus mykiss*), whereas feeding 50% will affect palatability and the feeding rate [56]. The present study’s results indicate that using yeast as a feed for *G. columna* does not significantly increase growth, which may be related to the influence of yeast cell walls, lignocellulosic biomass, and the most abundant carbohydrate at the time of coral digestion [57,58]. Kim et al. (1998) [59] contended that tough cell walls are the main constraint on yeast’s use as an aquaculture feed. Therefore, corals’ ability to digest live yeast or absorb sugars may require further research.

According to the nutritional research on microalgae, I and N are rich in DHA and EPA, respectively [60,61]. Living microalgae constituents such as PUFAs, vitamins, sterols, and carbohydrates have key nutritional value [62]. Factors influencing the bioutilization of microalgae include its size, shape, digestibility, biochemical composition, enzyme activity, and toxins as well as the target organism’s requirements for feeding [63]. Microalgae grown to the late logarithmic growth phase typically contain 10–20% lipid, 5–15% carbohydrate, and 30–40% protein [64]. Our results indicated no significant increase in the protein, lipid, or glucose content of *G. columna* specimens after 8 weeks of being fed I or S microalgae. The lipase and amylase content of the corals were also low, which may have led to an inability to metabolize fat and starch, thereby limiting the main cause of growth. In addition, the cell wall of microalgae generally comprises cellulose, which presents a challenge to bioaccessibility because of its low digestibility [61]. The microalgae I has cell diameter of approximately 3–5 μm, whereas the cell diameter of N is approximately 2–4 μm [65]. The mouth diameter of *G. columna* is 50–100 μm; therefore, *G. columna* can comfortably ingest these two feeds. Corals are coelenterates—whatever food they cannot digest after initial ingestion is excreted from their mouths. We observed microalgae in the coral’s body 1 h after eating, implying that the coral had swallowed the microalgae. Therefore, the results suggest that corals cannot digest microalgae after swallowing them, explaining the lack of significant increase in growth. Excessive feeding may cause excessive nutrients in the water and cause algae growth af-fects the growth of coral. Therefore, the feed selected in coral aquaculture should be one that can be quickly absorbed and does not pollute the water. During our study, the ammonia nitrogen and nitrite in the water were measured, and none of the experimental groups’ water contained more than the standard levels.

The 24-h observation of *G. columna* body composition and digestive enzyme activity (Figure 3) indicated that the protein content of the coral was highest at 12:00, measuring 486 ± 23.15 µg/mg, and was significantly lower at 18:00, 00:00, and 06:00, measuring 324.13 ± 21.03, 321.49 ± 19.46, and 345.21 ± 21.64 µg/mg, respectively. The digestive enzyme protease activity also exhibited marked variation throughout the day; at 6:00, 12;00, 18:00, and 00:00, its registered activity was 153.25 ± 20.32, 385.67 ± 16.48, 285.15 ± 17.12, and 167.85 ± 19.35 U/mg protein, respectively. The comparison of other aspects of digestive enzyme activity and protein content in the body (Figure 3 and Table 7.) indicated that low protein content occurs when digestive enzyme activity is low; when protein content is at its highest (12:00), the digestive enzyme activity is also its highest. Digestive enzyme activity was decreased at 18:00, at which point the protein content was also decreased, and finally when the digestive enzyme activity decreased to its daily low at 00:00, the body protein content was also at its lowest. Hence, the protein content increased to its highest level within 6 h of feeding, and the digestive enzyme activity decreased after 6 h, suggesting that the digestion time was within 6 h. According to Sebens and Koehl (1984) [66], digestion times vary among coral species. The digestion time of *Alcyonium siderium* fed zooplankton is approximately 4–6 h. The hydrocoral *Millepora complanata* reportedly completes digestion of its prey in approximately 24 h [12]. *G. fascicularis* begins to digest food 10 min after ingestion and finishes within 180 min [6]. Studies have failed to thoroughly determine the key role of light or food in corals’ physiological metabolism and growth. Light is a crucial factor in the physiology of coral symbiotic zooxanthellae and critical for the growth and maintenance of zooxanthellate coral in captivity [67,68]. Therefore, the digestion and absorption properties of corals change from day to day. When managing large-scale *G. columna* aquaculture, the establishment of definite feeding times is critical; we noted that the optimal feeding time for *G. columna* is between 6:00 and 12:00. At this time, higher protease activity aids digestion, metabolism, and absorption.

## 5. Conclusions

The coral aquaculture industry has gradually attracted attention, and feeding *G. columna* with the R(10) formulation can improve its growth and significantly improve its protein content compared with other feeds. The optimal feeding time is between 06:00 and 12:00, when the activity of digestive enzymes is highest and maximal benefit can obtained from the absorption of nutrients in ingested food. Our observations suggest that feeding with R can increase the number of coral polyps from 5 to 40 number over 2 months, accelerating the growth rate of *G. columna*. We expect our findings to be applied in coral farming enterprises. At present, the CITES-certified Taiwan Coral king Coral King Coral Farm (Kaohsiung, Taiwan) feeds corals with the R formulation between 6:00 and 12:00 daily, recording an annual output of up to 20,000 *G. columna* colonies, all housed in 150 × 60 × 30 cm tanks.

## Figures and Tables

**Table 1:** Nutrient composition of the various feeds.

Nutritional Indicators	R	S	I	N
Protein (µg)	76.67 ^a^ −1.56	37.00 ^d^ −1.33	66.00 ^b^ −1.33	49.00 ^c^ −0.67
Lipid (µg)	6.00 ^c^ −0.67	5.00 ^c^ −1.33	32.00 ^a^ −2	20.33 ^b^ −1.56
Glucose (µg)	22.00 ^b^ −0.67	29.00 ^a^ −0.67	0.63 ^c^ −0.16	0.67 ^c^ −0.11

R: artificial PUFA rich in animal protein; S: *Saccharomyces cerevisiae*; I: *Isochrysis galbana* tml; N: *Nannochloropsis oculata*. Different letters indicate significant differences among groups (*p* < 0.05). Values are expressed as means ± SDs (*n* = 3).

**Table 2:** Water quality conditions.

5%						10%			
Water Quality Conditions	C	R	S	I	N	R	S	I	N
Temperature (°C) Salinity (PSU) pHAmmonia nitrogen (mg/L) Nitrous acid (mg/L) Nitric acid (PPM) Calcium (PPM) Magnesium (PPM) Phosphate (PPM)	26.03 (0.24) 35.04 (0.22)	26.19 (0.14) 35.21(0.39)	26.20 (0.21) 35.12 (0.17)	26.32 (0.19) 35.21 (0.12)	26.31 (0.15) 34.22 (0.82)	26.21 (0.21) 35.21 (0.19)	26.03 (0.24) 35.12 (0.11)	26.47 (0.52) 34.20 (0.21)	26.93 (0.33) 35.04 (0.19)
	8.01 (0.31) 0.04 (0.01) 0.02 (0.01)	8.03 (0.21) 0.04 (0.02) 0.02 (0.01)	8.12 (0.42) 0.04 (0.03) 0.02 (0.01)	8.05 (0.13) 0.05 (0.02) 0.01 (0.01)	8.21 (0.42) 0.04 (0.03) 0.02 (0.01)	8.21 (0.93) 0.04 (0.02) 0.02 (0.01)	8.04 (0.21) 0.04 (0.04) 0.02 (0.01)	8.21 (0.48) 0.03 (0.01) 0.02 (0.01)	8.21 (0.24) 0.03 (0.01) 0.02 (0.01)
	0.20 (0.03) 409 (42.32) 1385(69.12)	0.18 (0.04) 415 (33.92) 1350(63.29)	0.30 (0.02) 410 (13.03) 1349 (31.03)	0.13 (0.02) 418 (20.03) 1320 (22.43)	0.05 (0.02) 424 (32.01) 1381 (53.22)	0.25 (0.08) 412 (34.21) 1351 (21.04)	0.20 (0.02) 427 (12.15) 1328 (31.26)	0.19 (0.04) 413 (30.21) 1365 (23.41)	0.20 (0.03) 410 (30.22) 1350 (30.34)
	0.02 (0.01)	0.02 (0.01)	0.02 (0.01)	0.02 (0.01)	0.02 (0.01)	0.02 (0.01)	0.02 (0.01)	0.02 (0.01)	0.02 (0.01)

Values are expressed as mean ± standard deviation (SD; *n* = 56 days); C: no feeding; R: artificial polyunsaturated fatty acid (PUFA) rich in animal protein; S: *Saccharomyces cerevisiae*; I: *Isochrysis galbana* tml; N: *Nannochloropsis oculate*.

**Table 3:** Body composition of corals 1 h after being fed different aquaculture feeds.

Nutritional Indicators	Feeding				
	C	R	S	I	N
Protein (µg/mg)	321.52 ^b^ −11.51	430.45 ^a^ −12.3	322.05 ^b^ −11.22	332.35 ^b^ −13.12	325.43 ^b^ −13.2
Lipid (µg/mg)	1.10 ^b^ −0.15	1.20 ^b^ −0.11	1.40 ^b^ −0.12	3.11 ^a^ −0.21	2.85 ^a^ 0.14
Glucose (µg/mg)	1.11 ^c^ −0.06	3.93 ^b^ −0.31	4.82 ^a^ −0.06	1.15 ^c^ −0.12	1.14 ^c^ −0.09

C: no feeding; R: artificial polyunsaturated fatty acid (PUFA) rich in animal protein; S: *Saccharomyces cerevisiae*; I: *Isochrysis galbana* tml; N: *Nannochloropsis oculata*. Different letters indicate significant differences among groups (*p* < 0.05). Values are expressed as means ± SDs (*n* = 30 colonies).

**Table 4:** Polyp number, zooxanthellae density, and chlorophyll a concentration of *G. columna* after 8 weeks of daily feeding.

Treatments	Feed	Zooxanthellae (Cells×10^7^ m^2^)	Chlorophyll a (µg/cm^2^)	Initial Polyps (Number ± 95%)	End Polyp (Number ± 95%)	Net Increase (Number ± 95%)
5%	C	3.71 (0.17)	53.22 (1.04)	5.00 (0.00)	33.00 (1.37)	28.00 (1.37)
	R	4.05 (0.71)	54.29 (1.23)	5.00 (0.00)	40.00 (2.34)	35.00 (2.34)
	S	3.79 (1.43)	53.73 (1.17)	5.00 (0.00)	32.67 (2.22)	27.67 (2.22)
	I	3.98 (0.94)	53.98 (1.39)	5.00 (0.00)	34.00 (0.67)	29.00 (0.67)
	N	4.12 (1.02)	54.05 (0.28)	5.00 (0.00)	32.33 (1.56)	27.33 (1.56)
10%	R	3.93 (1.76)	54.02 (1.32)	5.00 (0.00)	47.33 (0.89)	42.33 (0.89)
	S	4.21(1.53)	53.69 (1.75)	5.00 (0.00)	35.35.00 (2.00)	30.00 (2.00)
	I	4.27(0.92)	54.38 (1.43)	5.00 (0.00)	36.00 (0.67)	31.00 (0.67)
	N	4.36(1.47)	54.64 (0.00)	5.00 (0.00)	32.67 (1.78)	27.67 (1.78)

C: no feeding; R: artificial polyunsaturated fatty acid (PUFA) rich in animal protein; S: *Saccharomyces cerevisiae*; I: *Isochrysis galbana* tml; N: *Nannochloropsis oculate*. Values are expressed as means ± SDs (*n* = 30 colonies).

**Table 5:** Body composition of *G. columna* after 8 weeks of feeding with different aquaculture feeds.

Nutritional Indicators	Treatments								
	C	R		S		I		N	
		5% (I)	10% (II)	5% (I)	10% (II)	5% (I)	10% (II)	5% (I)	10% (II)
Protein (µg/mg)	374.73 ^b^ (11.50)	474.01 ^a^ (23.00)	486.78 ^a^ (36.41)	327.72 ^b^ (21.80)	383.18 ^b^ (12.60)	386.87 ^b^ (12.60)	385.57 ^b^ (19.80)	339.23 ^b^ (12.14)	321.79 ^b^ (8.85)
Lipid (µg/mg)	1.96 ^b^ (0.15)	2.85 ^a^ (0.13)	3.02 ^a^ (0.29)	1.65 ^b^ (0.15)	1.72 ^b^ (0.10)	1.98 ^b^ (0.12)	2.13 ^b^ (0.21)	1.69 ^b^ (0.42)	1.64 ^b^ (0.14)
Glucose (µg/mg)	1.11 (0.06)	1.28 (0.20)	1.32 (0.22)	1.13 (0.07)	1.26 (0.06)	1.22 (0.09)	1.15 (0.12)	1.13 (0.07)	1.11 (0.09)

C: no feeding; R: artificial PUFA rich in animal protein; S: *Saccharomyces cerevisiae*; I: *Isochrysis galbana* tml; N: *Nannochloropsis oculata*. Different letters indicate significant differences among groups (*p* < 0.05). Values are expressed as means ± SDs (*n* = 30 colonies).

**Table 6:** Activity of digestive enzymes in *G. columna* after 8 weeks of feeding with different aquaculture feeds.

Test Items	Treatments								
	C	R		S		I		N	
		5% (I)	10% (II)	5% (I)	10% (II)	5% (I)	10% (II)	5% (I)	10% (II)
Protease (U/mg protein)	214.37 ^b^ (11.22)	324.67 ^a^ (26.68)	325.70 ^a^ (34.88)	206.24 ^b^ (7.31)	196.66 ^b^ (12.13)	182.13 ^b^ (42.85)	168.68 ^b^ (30.17)	191.75 ^b^ (35.94)	190.17 ^b^ (33.92)
Lipase (U/mg protein)	10.01 (0.80)	12.16 (0.52)	12.84 (0.43)	10.04 (0.39)	10.13 (0.37)	9.42 (0.64)	10.30 (0.42)	9.09 (1.30)	9.42 (0.64)
Amylase (U/mg protein)	1.47 (0.08)	1.45 (0.15)	1.61 (0.24)	1.38 (0.43)	1.17 (0.54)	1.27 (0.08)	1.26 (0.06)	1.18 (0.52)	1.26 (0.58)

C: no feeding; R: artificial PUFA rich in animal protein; S: *Saccharomyces cerevisiae*; I: *Isochrysis galbana* tml; N: *Nannochloropsis oculata*. Different letters indicate significant differences among groups (*p* < 0.05). Values are expressed as means ± SDs (*n* = 30 colonies).

**Table 7:** Changes in digestive enzymes of R-fed *G. columna* over a 24-h period.

Test Items	Time (Hour)			
	6	12	18	24
Protease U/mg protein	153.25 (20.32) ^c^	385.67 (16.48) ^a^	285.15 (17.12) ^b^	167.85 (19.35) ^c^
Lipase U/mg protein	4.01 (0.08) ^c^	10.16 (0.52) ^a^	9.06 (0.42) ^b^	4.39 (0.12) ^c^
Amylase U/mg protein	0.95 (0.21)	1.29 (0.11)	1.17 (0.08)	1.04 (0.16)

Different letters indicate significant differences among groups (*p* < 0.05). Values are expressed as means ± SDs (*n* = 30 colonies).

## Data Availability

Not applicable.
